# A Portable Farmland Information Collection System with Multiple Sensors

**DOI:** 10.3390/s16101762

**Published:** 2016-10-22

**Authors:** Jianfeng Zhang, Jinyang Hu, Lvwen Huang, Zhiyong Zhang, Yimian Ma

**Affiliations:** College of Information Engineering, Northwest A&F University, Yangling 712100, China; xnhujy@126.com (J.H.); zzy@nwsuaf.edu.cn (Z.Z.); mym@nwsuaf.edu.cn (Y.M.)

**Keywords:** agriculture information collection, multiple sensors, chlorophyll relative content, CDMA

## Abstract

Precision agriculture is the trend of modern agriculture, and it is also one of the important ways to realize the sustainable development of agriculture. In order to meet the production requirements of precision agriculture—efficient use of agricultural resources, and improving the crop yields and quality—some necessary field information in crop growth environment needs to be collected and monitored. In this paper, a farmland information collection system is developed, which includes a portable farmland information collection device based on STM32 (a 32-bit comprehensive range of microcontrollers based on ARM Crotex-M3), a remote server and a mobile phone APP. The device realizes the function of portable and mobile collecting of multiple parameters farmland information, such as chlorophyll content of crop leaves, air temperature, air humidity, and light intensity. UM220-III (Unicore Communication Inc., Beijing, China) is used to realize the positioning based on BDS/GPS (BeiDou Navigation Satellite System, BDS/Global Positioning System, GPS) dual-mode navigation and positioning system, and the CDMA (Code Division Multiple Access, CDMA) wireless communication module is adopted to realize the real-time remote transmission. The portable multi-function farmland information collection system is real-time, accurate, and easy to use to collect farmland information and multiple information parameters of crops.

## 1. Introduction

With the continuous development of information technology, it is used to realize the transformation of traditional agriculture to the precise and intensive informationized agriculture, which is system engineering used to maintain the sustainable development of agriculture and is the tendency in modern agriculture technology. Therefore, the application of information technology in agriculture is bound to be one of the key research areas in modern agricultural engineering.

In order to meet the production requirements and the efficient use of agricultural resources, modern information technology is used to collect some necessary farmland information in the crop growth environment, which is the foundation of modern agriculture. Early farmland information collection systems are mostly based on PC, which have high cost and high power consumption. With the development of electronics and communication technologies, the new generation of information technology represented by 3S (GIS, GPS, and RS), Internet of things, cloud computing, and big data has been widely used in all kinds of agriculture fields, which promotes the development of agricultural modernization [1,2,3,4,5,6,7,8,9]. The collection, transmission, storage, and processing of farmland information are the basis of the development of modern agriculture. Du et al. [2] proposed an approach to evaluation of the agricultural environment from on-farm sites to regional scales, and designed a regional-scale monitoring system by integrating with the spatial analysis technology of WebGIS. In the monitoring system, three types of functions were implemented, including site data positioning, regional-scale evaluation, and thematic mapping. Chen et al. [3] developed a portable agricultural information collection system based on cross-platform mobile GIS, which could capture the GPS coordinates of the farmland, the agricultural attribute data, image information, and sent them to the monitoring system immediately using 3G network or GPRS network. Internet of things is a network system which integrates the functions of sensing, transmission, and intelligent decision and control, which provides a good solution for the farmland information collection. Hirofumi et al. [7] proposed a reliable wireless hydroponic liquid supply control system for hydroponic tomato cultivation with no data loss by using the 400 MHz wireless band and the IEEE 802.15.6 standard. Yao and Zhou [10] designed a farmland information collection system based on RFID (Radio Frequency Identification, RFID) and GPS (Global Positioning System, GPS), which took MSP430F5438 (Texas Instruments, Dallas, TX, USA) as the main controller and realized the collection and processing of soil temperature and humidity, light intensity, CO_2_ concentration, and achieved the collecting position and time through the GPS module. Guo et al. [11] designed a farmland environment monitoring network system based on ZigBee, which had good real-time function and high accuracy, and effectively solved some problems in large-field environment monitoring such as complex wiring, high cost, and small communication range. Modern internet of things technologies have been effectively improving the accuracy and real-time of agricultural data collection. However, crop agriculture has characteristics of wide area, big data, being significantly seasonal and highly cyclical, and fixed-point measurement has some disadvantages of complex wiring, lots of running maintenance, and poor flexibility. Hence, we designed a portable multifunction field information collection system to meet timely and flexible application requirements.

In this paper, the farmland information collection system based on STM32 (a 32-bit comprehensive range of microcontrollers based on ARM Crotex-M3) is developed, which realizes the function of portable and mobile collecting of multiple parameters information, such as chlorophyll content of crop leaves, air temperature, air humidity, and light intensity. This paper firstly introduces the composition of the farmland information collection system, which consists of three parts of the farmland information collection device, the remote server, and the mobile phone app. Then, in Section 2, the structure and design of the portable multi-function farmland information collection devices are described in detail. Section 3 describes the related experiments, and the experimental results are analyzed and evaluated. The portable multi-function farmland information collection system can collect the farmland information and multiple parameters information of crops, which are low cost, timely, accurate, and easy to use, and can be good to make up for the lack of the fixed-point measurement.

## 2. Design of the Portable Farmland Information Collection System

Portable farmland information collection system is one approach to achieve timely and effective measurement of multi-parameter information in a small agricultural environment. This section describes the system structure, design, and implementation of the farmland information collection device.

### 2.1. Overview of the Portable Farmland Information Collection System

**Figure 1:**
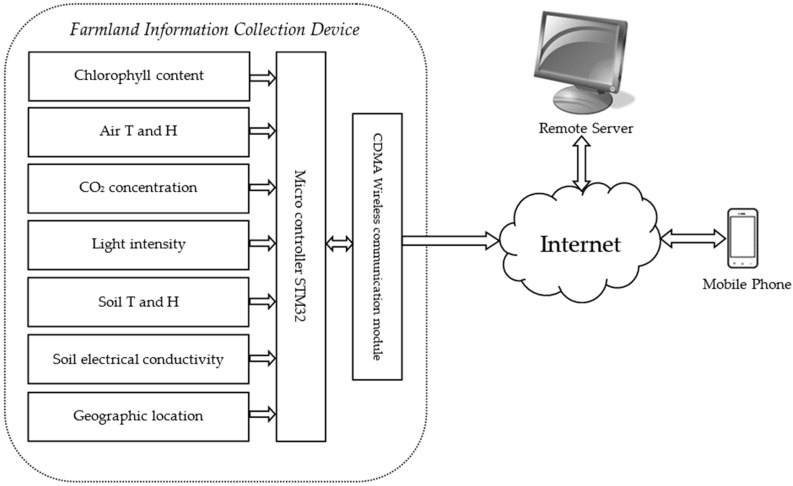
Scheme of the farmland information collection system.

The system scheme of farmland information collection system is shown in Figure 1, which is composed of three parts, including the farmland information collection device, the remote server and the mobile phone app. The farmland information collection device firstly collects environmental information and crop growth information, and uploads them to the remote server; then the software running on the remote server parses, displays, and stores the collected information, and sends it to a PC or mobile phone app; finally, the environment monitoring system running on the PC receives and stores the data to provide detailed and accurate basic data for the automation management of agricultural production. The managers can remotely monitor the farmland data and issue timely responses and processing through a mobile phone app. 

### 2.2. Farmland Information Collection Device Based on STM32

**Figure 2:**
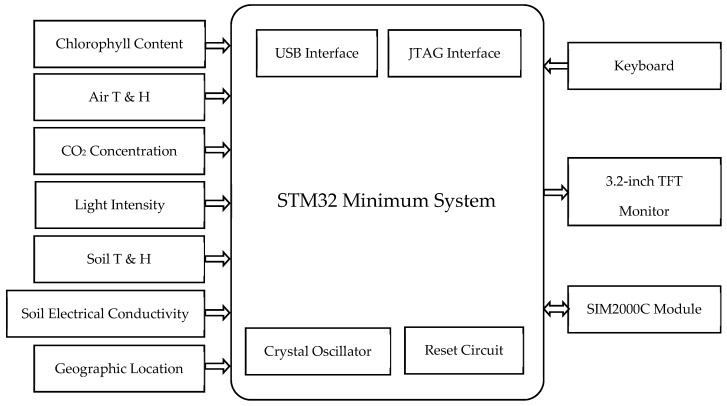
Block diagram of the farmland information collection device.

The portable multi-function farmland information collection device can collect, display, and transmit farmland information. The block diagram is given in Figure 2. The system is cored with a 32-bit microcontroller STM32F103VET6 (STMicroelectronics, Dallas, TX, USA). The operation system adopts μC/OS II. The μC/GUI is ported to realize the control for 3.2-inch TFT monitor. According to the application requirements, the system realizes the measurement of multiple parameters information, such as chlorophyll content of crop leaves, air temperature, air humidity, light intensity, location information, CO_2_ concentration, soil temperature, soil moisture. The basic requirements for the measurement parameters are shown in Table 1. A 3.2-inch TFT monitor is selected as the display module. The system uploads the collected farmland information to the remote server through SIM2000C, a CDMA (Code Division Multiple Access, CDMA) wireless data transmission module.

### 2.3. Software Structure of the Collection Device

The farmland information collection device realizes the function of measurement of farmland information, collection of location information, and wireless data transmission. The operation system adopts μC/OS II V2.86, which is a real-time operation system, divides the system tasks to some different subtasks and reasonably sets the different priority of the subtasks, to realize stable real-time operation.

**Figure 3:**
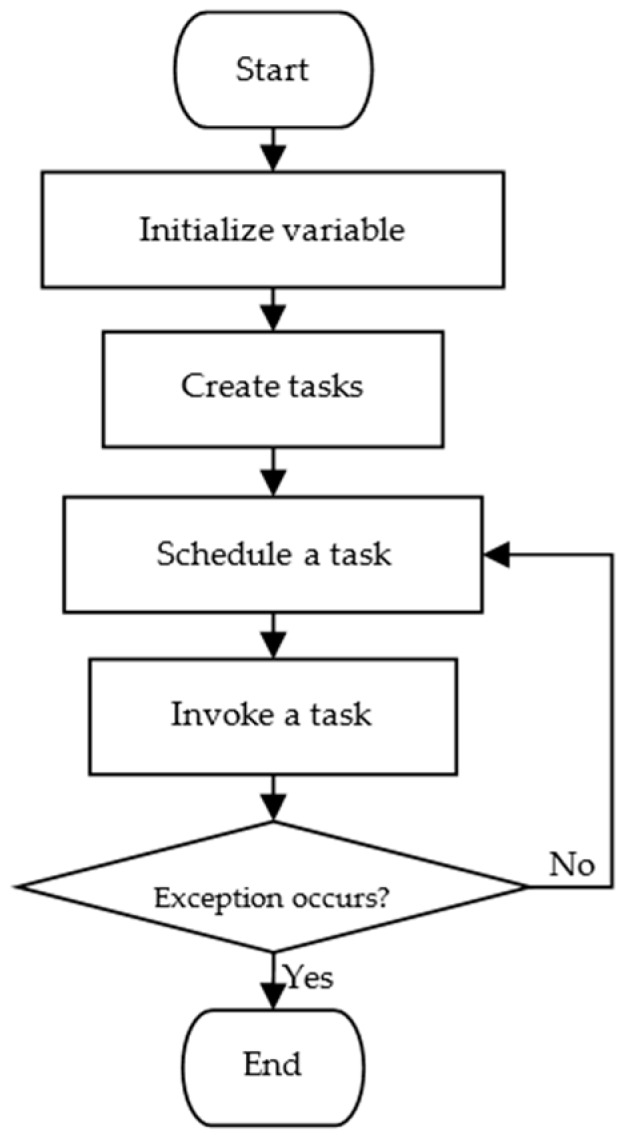
Operation flowchart of the farmland information collection device.

The system tasks are divided into 11 subtasks, including the measurement of chlorophyll relative content; the measurement of environmental temperature and humidity; the measurement of light intensity; the measurement of CO_2_ concentration; the measurement of soil temperature, humidity, and electrical conductivity; the parsing of positioning data; CDMA wireless data transmission; display; serial data storage; key detection; and idle task. The higher priority tasks have a higher real-time requirement. The lower priority tasks being performed can be interrupted by any high priority tasks, which ensures the real-time performance of the system and makes full use of the system resources. The flowchart of the system is shown in Figure 3.

### 2.4. Rapid and Non-Destructive Detection Technology of Chlorophyll Content of Crop Leaves

The content of chlorophyll is a good indicator of plant nutrition, photosynthesis, and growth conditions. Collecting real-time and accurate crop chlorophyll content of crop leaves is the basis of crop nutrition diagnosis and scientific fertilization management. It is also one of the key technologies in the implementation of precision agriculture. Spectrum detection technology can quickly and accurately obtain the growth and nutritional status of the crop, which can give tips for fertilization, irrigation, and applying pesticides for the growth of crops. In recent years, the method of measurement of chlorophyll relative content based on multi-spectrum has attracted the attention of many researchers, which has applied to many crops, including rice nitrogen fertilizer recommendation [12,13], potato [14,15], wheat [16,17], and corn [18]. Zhang et al. [19] proposed the quantitative analysis model for the relation between the leaf relative chlorophyll content and the reflectance spectra through researching the non-destructive testing of leaf chlorophyll content of winter wheat, and preprocessing using MSC and SNV improved the accuracy. Liu et al. [20] pointed out that when the plant was in the process of photosynthesis, it mainly absorbed red light and blue light from the sun; two wavelengths of light selected as the characteristic wavelengths light sources were the infrared region (650 nm) and near-infrared region (940 nm); the chlorophyll content of leaves was determined by comparing the light intensity of the reflected light and the transmitted light through the leaves. SPAD is defined as

(1) $$SPAD=K\mathrm{lg}\left(\begin{array}{c} \underline{IR_{t}/IR_{0}}\\ R_{t}/R_{0} \end{array}\right)$$

where *IR_t_* is the light intensity of near-infrared light at the wavelength of 940 nm through the crop leaves; *IR*_0_ is the light intensity of near-infrared light source at a wavelength of 940 nm; *R_t_* is the light intensity of red light at the wavelength of 650 nm through the crop leaves; *R*_0_ is the light intensity of red light source at a wavelength of 650 nm; *K* is a scaling factor.

**Figure 4:**
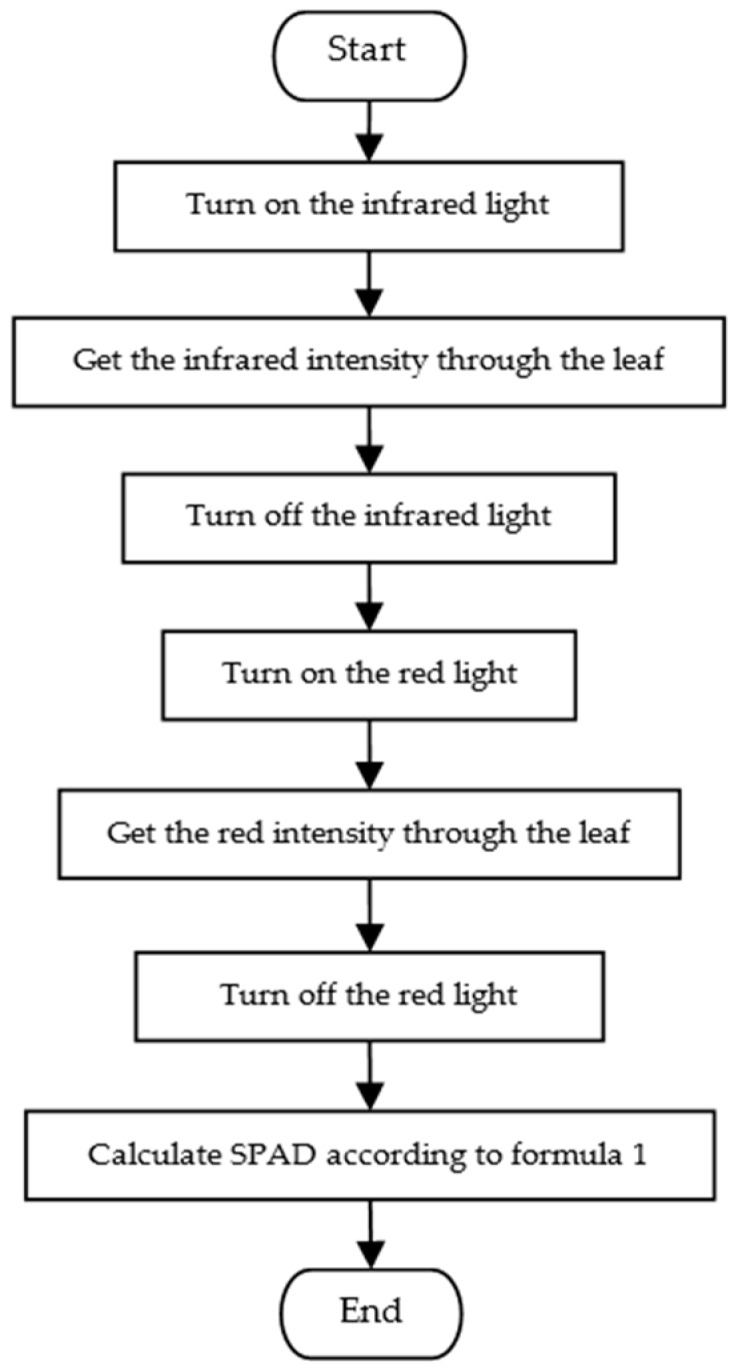
Program flowchart of Chlorophyll measurement module.

The chlorophyll measuring module consists of two parts, including the light emitting circuit and the light intensity detection circuit, the flowchart is shown in Figure 4. Light intensity detection circuit adopts OPT101 sensor, which integrates a photosensitive device and a signal amplifier, and the output voltage is linearly dependent on the light intensity; A/D module adopts ADS7841, which has 12-bit resolution, and the accuracy meets the measurement requirements.

### 2.5. Farmland Environment Information Collection

Temperature and humidity are important indicators that can have direct impact on crop growth. Environmental temperature is the drive force to accelerate the growth of plants, which has a direct impact on plants’ growth, and appropriate temperature will accelerate the growth of plants. Humidity refers to the content of moisture in the air, and inappropriate humidity is not only conducive to the growth of plants because it cannot meet the needs of plant photosynthesis, but also because it can cause pest problems. Light will directly affect the photosynthesis of plants, thereby affecting the development and growth of plants, production of crops, CO_2_ concentration, and humidity. CO_2_ concentration is an important indicator for plant growth, which can directly affect the photosynthesis of plants. If the CO_2_ concentration is too low, photosynthesis rate of plants will slow down, and a sharp slowdown affects plants’ growth and development. If the CO_2_ concentration is too high, the stomata of plants would shut down, the conductance would reduce a lot, and photosynthesis, respiration and transpiration of plants will suffer a lot. Therefore, only the most suitable CO_2_ concentration is conducive to plant growth and development.

The AM2302 sensor is used to collect the temperature and humidity. AM2302 is a humidity-sensitive capacitance digital temperature and humidity sensor, which integrate high-precision temperature measurement devices and capacitive humidity sensing device. The temperature measurement range is from −40 °C to 80 °C, the resolution is 0.1 °C, and the accuracy is ±0.5 °C; humidity measuring range is from 0% to 99% RH, the resolution is 0.1% RH, and the accuracy is ±2% RH (at 25 °C ambient temperature). The GY-30 digital light intensity sensor is used to collect light intensity, and the measurement range is from 0 to 65,535 Lx. CO_2_ concentration is measured by MH-Z14A infrared sensor, which uses the principle of non-dispersive infrared that has built-in temperature compensation to detect the CO_2_ concentration in the environment, the measuring range is from 0 to 5000 ppm, and the measurement accuracy is ±(50 ppm + 3%).

### 2.6. Soil Information Collection

Soil temperature directly affects the formation and texture of soil, and the growth of the crop, and too high or too low of soil temperature will do harm to the agricultural production. Soil moisture is an important indicator to measure the soil water content. If the soil moisture is too low, there would be several consequences, for example, the soil would be arid, photosynthesis of crops would be hampered, the yield and quality of crops would be affected and even the crop would wither or die; if the soil moisture is too high, there would be some other consequences, for example, it would hinder soil permeability and the soil microbial activity would suffer a lot, and it would impede the breathing of crops and cause lodging, leggy crops and other issues.

The measurements of soil moisture, soil temperature and soil conductivity use the sensor of SMET-2. SMET-2 sensor is a soil performance sensor that is based on dielectric technology theory, which uses dual frequency technology and is set of soil moisture, soil temperature, and soil conductivity, has the algorithm of humidity compensation, temperature compensation and conductivity compensation, and connects with the microcontroller through RS-485 interface. Microcontroller uses MODBUS-RTU protocol to communicate with SMET-2, and the specific parameters are shown in Table 2.

### 2.7. Field Positioning Technology

The UM220-III chip uses BDS/GPS (BeiDou Navigation Satellite System, BDS/Global Positioning System, GPS) dual-mode system for joint positioning, and the parameters of the chip are shown in Table 3.

**Figure 5:**
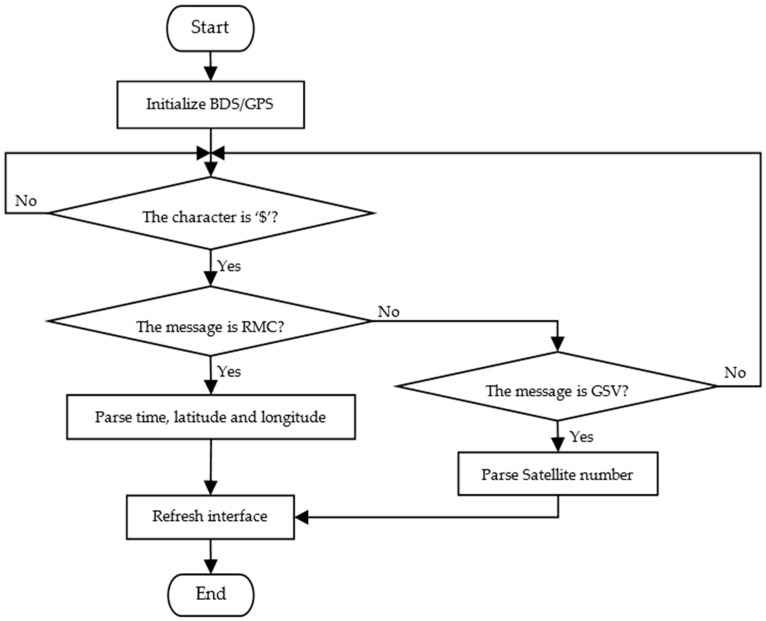
Parsing flowchart of obtaining real-time location information, $ is the beginning character of all the messages, RMC provides the minimum recommended data message, and GSV describes the visible satellite messages.

The Beidou positioning mode uses the active positioning principle. The response signal is emitted by the user, and the position information can be collected from the central station, and the central station on the ground transmits the calculated location information to the user via satellite. The UM220-III chip adopts the GNSS (Global Navigation Satellite System, GNSS) multi-system integration and Kalman filtering algorithm to guarantee to have continuous reliable positioning results in a variety of complex environments, and the positioning accuracy is up to 2.5 m CEP. The data interface adopts NMEA-0183 protocol, which is widely used in field vehicle monitoring, navigation, telecommunications, handheld devices, timing electricity, and other areas. UM220-III has a low power consumption, which is suitable for power-critical portable mobile applications. STM32 system can resolve the latitude, the longitude, real time, the number of real-time satellites, and so on. The flowchart of obtaining real-time location information is shown in Figure 5. The system can compare the above information with the farmland parameters obtained from the field information collection device, which can help agricultural technicians to have access to the health conditions and environmental information of crops in different fields in real time. In the Unicore protocol of UM220-III, all input and output statements are referred to messages. Each message is a string with some ASCII characters. All messages start with a character of ‘$’. GSV message describes the visible satellites, each GSV message contains the information of no more than four satellites. The format of the GSV message is ‘$--*GSV*, *NoMsg*, *MsgNo*, *NoSv*, *sv1*, *elv1*, *az1*, *cno1*, *sv2*, *elv2*, *az2*, *cno2*, *sv3*, *elv3*, *az3*, *cno3*, *sv4*, *elv4*, *az4*, *cno4*’. RMC message provides the minimum data recommended, including date, time, latitude, longitude, and so on. The format of the GSV message is ‘$--*RMC*, *time*, *status*, *Lat*, *N*, *Lon*, *E*, *spd*, *cog*, *date*, *mv*, *mvE*, mode*cs’. For a detailed definition of the parameters of GSV and RMC, please see in the Reference [21].

### 2.8. Wireless Data Transmission Technology

There are three common methods of data communication: one is wired methods, mainly including RS-485 bus, CAN bus, and network connection; the second is short distance wireless communication mode, including ZigBee, infrared, and Bluetooth; the third is long distance wireless communication mode, mainly including GSM, GPRS, and CDMA communication. Because of the complex wiring and higher cost, the wired connection is less used in the field of agriculture. Short distance wireless communication mode cannot meet the data transmission distance and the high dispersion of the agricultural field. The communication methods commonly used in agricultural areas, are mainly based on mobile phone network, such as GSM, GPRS, CDMA and other communications, which have a wide range of transmission distance, great real-time ability, and other characteristics.

SIM2000C, produced by Simcom, is a low power consumption, fast CDMA module, its peak current is 1.0 A, and it is more suitable for portable devices. When the operating frequency is 800 MHz, its maximum rate is up to 153.6 kbps. The module parameters are shown in Table 4.

The system uses SIM2000C’s data transmission service, namely CDMA2000 1×. It receives commands from the STM32 serial port to set module of wireless communication. The communication uses the socket communication mode based on TCP/IP protocol; the client software runs on the server side, which is always listening to the local specific network ports; once it receives the data uploaded from CDMA wireless data transmission module, it will parse and display each frame of data in a predetermined data format and store the data to the local machine according to the fixed format so that the user can query and process the data.

### 2.9. Power Supply Circuit

**Figure 6:**
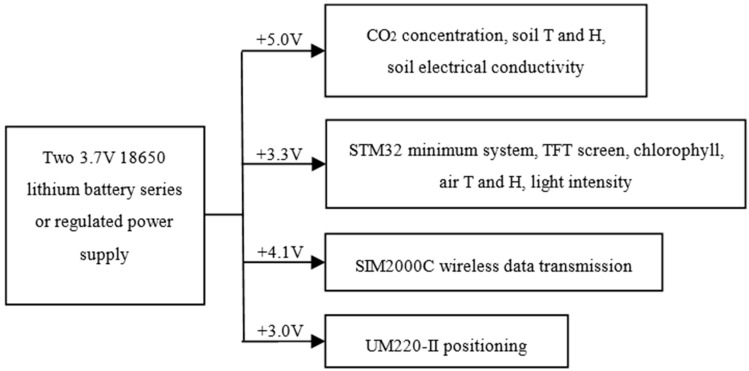
Block diagram of the power supply system.

The collection instrument needs four kinds of voltage sources of +5 V, +4.1 V, +3.3 V, and +3.0 V. In order to ensure the stable and reliable running of the system, the power supply adopts two 18,650 rechargeable lithium battery in series, with a rated voltage of 3.7 V and a capacity of 2900 mA each cell. Then, the power supply regulates the input voltage to +5.0 V through the LM2596-5.0 for CO_2_ concentration sensor, the soil temperature, and humidity module; regulates the + 5 V to 3.3 V through AMS1117-3.3V for STM32 system, TFT display screen, chlorophyll measurement circuit, environmental temperature and humidity, and light intensity module; regulates the input voltage to +4.1 V through mic29302 for SIM2000C module; regulates the input voltage to +3.0 V through LM317 for UM220-III module. The system power supply block diagram is shown in Figure 6.

### 2.10. The Design of System Stability

According to the needs of the system design, we take some protective and anti-interference measures to ensure the system’s normal, stable running. They are summarized below:

(1) Power supply adopts a self-recovery fuse with a capacity of 2 A for the overcurrent, overheating protection. The traditional fuse needs to be replaced after the trigger once, but the self-recovery fuse can automatically recovery after a troubleshooting.
(2) The circuit is connected in series with a SS34 reverse polarity protection diode. When the connection of the power’s positive and negative electrode is wrong, it can disconnect the circuit to protect the system circuit, only after a proper connection can the circuit normally run.
(3) The power supply and the main components are designed with two decoupling capacitances with a capacity of 0.1 µF and 100 µF for improving the power supply ripple and improving the stability of the power supply. Among them, the 0.1 µF capacitor is used to filter high frequency signal interference. The 100 µF capacitor plays the role of buffer and stability, which can reduce the impact on the rear circuit, so that the voltage does not have too much volatility and can ensure the stability of the circuit.
(4) Power supply uses SMBJ5.0A transient suppression diode to suppress the power of instantaneous pulse, such as electrostatic discharge effect, surge and power supply noise, etc.
(5) On the RUIM card, we use SMF05C to do electrostatic protection, which is used to improve the ability to anti-interference to protect the RUIM card. In addition, five TVS diodes are integrated to the SMF05C, which effectively remove the interference.
(6) On the RXD, TXD of SIM2000C, we do the processing of compatibility. Because the STM32 operating voltage is 3.3 V, and the voltage of SIM2000C is 2.85 V, after compatibility processing, the module can be compatible with 3.3 V/5 V system.
(7) Power input terminal of each module is designed with a resistor of 0 ohm. After the circuit is fine, switch on the circuit, which can avoid the circumstance that the system fault is too complex to exclude.
(8) When drawing PCB, according to the package of the components, we take reasonable layout and wiring, and fully take into account the system’s anti-disturbance and stability.

The portable multi-function farmland information collection device, which is cored with a 32-bit microcontroller STM32F103VET6 and the operation system of μC/OS II, can collect multiple field information, such as chlorophyll content of crop leaves, air temperature, air humidity, light intensity, and location information. This device also could measure CO_2_ concentration, soil temperature, and soil humidity moisture with some external sensors. The total cost of the hardware is not more than 150 dollars.

## 3. System Evaluation

According to the requirements of the National Key Technology R&D Program of China (No. 2012BAH29B04) and the Fundamental Research Funds for the Central Universities, Northwest A&F University, Xianyang, China (No. 2014YB068), the system was developed to collect the field crop information in a timely manner. The measuring results were analyzed and compared with some commonly used single-function instruments. After the system is realized, the function and performance of the system are tested. This section describes the test platform and related experiments, and the results are analyzed and evaluated.

### 3.1. Test Platform

PC: HP Pro 3380 MT with Intel Pentium Dual Core G2020, 4 GB DDR3 memory, and 500 GB 7200 RPM hard disk.

Experimental instrument: A portable farmland information collection device, named by MGT2.0, with functions of positioning, CDMA data transmission and the measurement of crop leaves’ chlorophyll content, environmental temperature, humidity, and light intensity.

Measuring instruments for the accuracy comparison and analysis: The crop leaves’ chlorophyll content is compared by SPAD-502 Chlorophyll Meter produced by Minolta Konica Inc., Tokyo, Japan, and the measurement accuracy is ±1.0 SPAD under normal temperature environment. The environment temperature and humidity are compared by AR837 digital temperature and humidity instrument, which was produced by SMART SENSOR, Hong Kong, China, the temperature measurement range is −10–50 °C, and the measurement error is ±3%; The humidity measurement range is 5%–98% RH, and the measurement error is ±5%. The light intensity is compared by DT-1308 illumination meter, which is produced by Shenzhen Huashengchang (CEM), Shenzhen, China, the measuring range is 40–40,000 Lx, and the precision is ±3.5% + 20 d.

Experimental environments and requirements: The experimental environments were the Information Engineering Laboratory of College of Information Engineering, Northwest A&F University, Xianyang, China, and three different classic arid and semi-arid agriculture areas surrounding Yangling town of Shaanxi Province in China, including the classic Arid and Semi-Arid Agriculture Institute of China, Juliang Farm and the demonstration field in Rougu Town. The continuous running experiments were in the laboratory. The field experiments selected the classic growth stages for wheat and corn for collecting the farmland and crop information.

### 3.2. Basic Experiments

**Figure 7:**
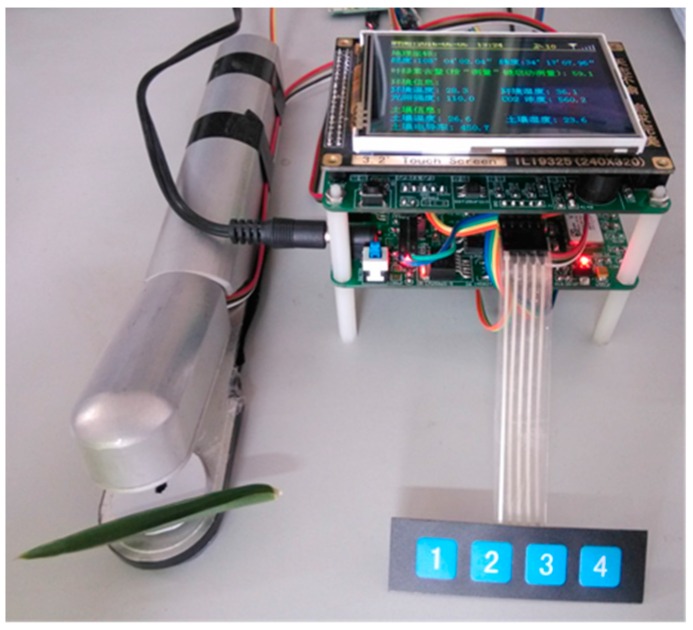
The experiment instrument of the farmland information collection device.

**Figure 8:**
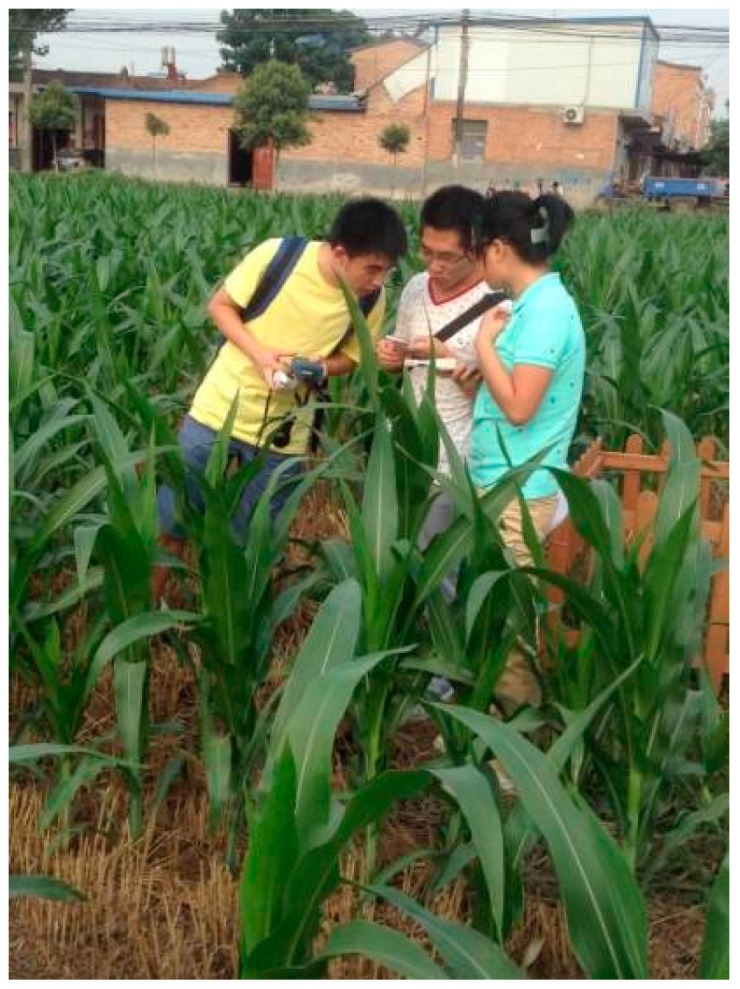
Farmland experiment.

Some experiments to test the function, stability, and reliability of the system, include more than 48 h of continuous running in the laboratory, mobile measurement, and some farmland applications. Figure 7 shows the farmland information collection device. Figure 8 shows one of the pictures in the farmland experiments.

#### 3.2.1. Test of Farmland Information Collection Device

After powering the system, the screen shows “please hold the chlorophyll clip”, which achieves the light intensity measurements of red light and near-infrared light under no-load conditions to complete the initialization calibration. When collecting the chlorophyll relative content, the leaf needs to be clamped in the clip, and press the “chlorophyll measurements” button to start a measurement, the results will be displayed on the screen. When collecting the soil information, select an appropriate measurement point away from the stones and other hard areas, remove the surface of sand soil and keep the loose degree of the soil underlying surface, vertically and completely insert the measurement probe in the soil, and avoid sloshing the probe.

**Figure 9:**
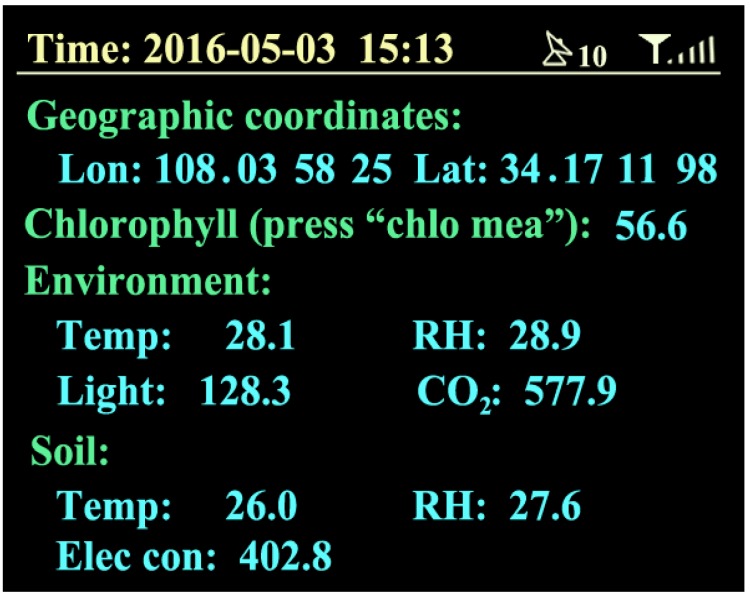
Sample of farmland information—on 3 May 2016, at 15:13—including with the geographic coordinates by Lon (longitude, degree) and Lat (latitude, degree), Chlorophyll (chlorophyll relative content, SPAD), Environment information by the Temp (air temperature, °C), RH (air humidity, %), Light (light intensity, Lx), and CO_2_ (CO_2_ concentration, ppm) and Soil information by the Temp (soil temperature, °C), RH (soil humidity, %), and Elec con (soil electrical conductivity, µs/cm).

After a good placement of all sensors, turn on the collection instrument. If it is set in the automatic mode, it will directly collect the location information, environmental temperature, environmental humidity, CO_2_ concentration, light intensity, soil temperature, soil moisture, and soil electrical conductivity. The farmland information collected will quickly display on the screen, and be uploaded to a remote server through the CDMA every 5 s. Figure 9 shows the real-time farmland information on 3 May 2016, at 15:13. If you set the automatic measurement function off, the user should start a farmland information measurement and upload data through the button. 

#### 3.2.2. Test of Wireless Data Transmission

For realizing the remote data transmission under the dynamic IP network environment, the domain name resolving software is used to establish the Internet host with a fixed domain name in the internal network. The Oray dynamic domain name resolving software can provide the internet access service through the domain name, which is regardless of the connection obtained by a dynamic or static IP. The system obtains the public domain name and port through the Oray software. Set the internal network address of 192.168.1.200, and use the port number 52525. After successful mapping, the Oray software allocates the only server address for the local machine with the domain name of “users.wicp.net”, and the port number 25422. Then the address is taken as the remote server address. When running the network debugging assistant to monitor the port 52525, real-time data will be shown. The “measure” button will trigger a chlorophyll relative content measurement, otherwise it will continuously maintain the previous measurement data.

The data in every frame starts from the characters of ‘*’, and the character of ‘#’ as a delimiter. After parsing, the parts are the uploading time, longitude, latitude, chlorophyll relative content, environmental temperature, environmental humidity, light intensity, CO_2_ concentration, soil temperature, soil moisture, and soil electrical conductivity respectively, such as a piece of ‘*2016-5-4 19:20:59#108.066978#34.286189#56.6#28.9 #30.3#60.0#591.0#26.7#26.2#476.0#’. After parsing it, the uploading time is 19:20:59 on 4 May 2016, longitude is 108.066978°, latitude is 34.286189°, chlorophyll relative content is 56.6 SPAD, environmental temperature is 28.9 °C, environmental humidity is 30.3% RH, light intensity is 60.0l×, CO_2_ concentration is 591.0 ppm, soil temperature is 26.7 °C, soil humidity is 26.2% RH, and soil electrical conductivity is 476.0 µs/cm.

#### 3.2.3. Test of Mobile Phone App

A mobile phone app is convenient to view the real-time farmland information, and when the collection device runs in the field, greenhouses, and other unattended circumstances for a long time, it can provide real-time remote monitoring of farmland information. 

**Figure 10:**
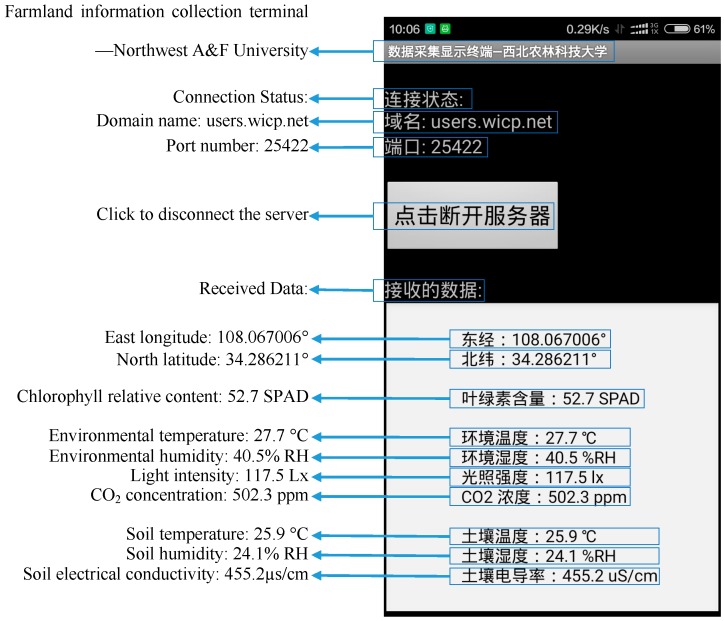
Interface of mobile phone APP in Chinese.

The specific operation steps are: turn on the network services of mobile phone; run the app; click the “connect server” button; the phone will take the initiative to connect to the remote server; after the successful connection, if the server receives the data from the farmland information collecting instrument, the client software running on the server would send the farmland information to the mobile phone; if the app receives the data, it would display them shown as Figure 10.

### 3.3. System Evaluation

**Figure 11:**
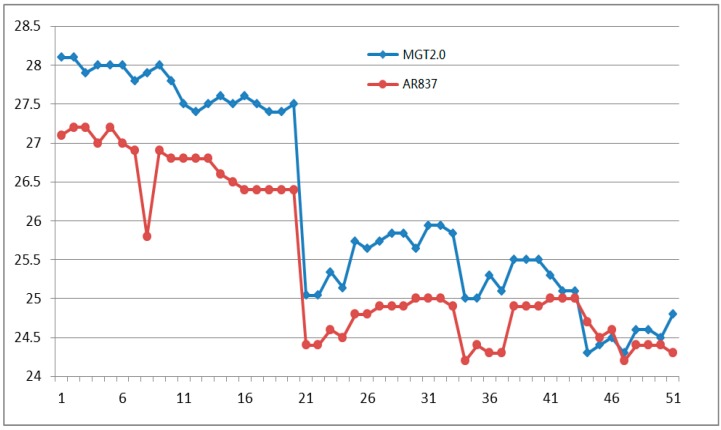
Fifty air temperature (°C) experimental results by MGT2.0 (marked by **blue** line) and AR837 (marked by **red** line).

**Figure 12:**
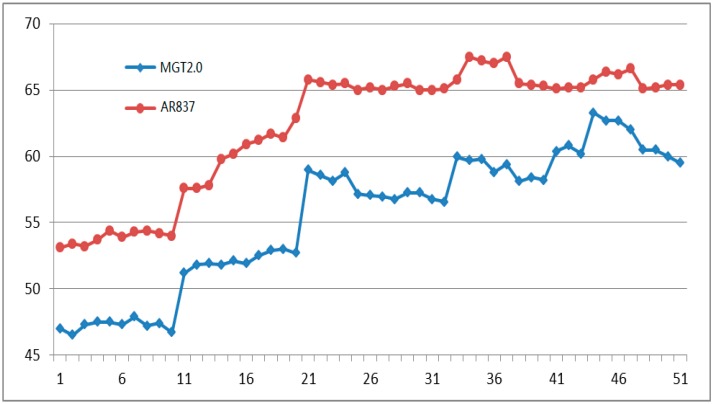
FIfty air humidity (%RH) experimental results by MGT2.0 (marked by **blue** line) and AR837 (marked by **red** line).

**Figure 13:**
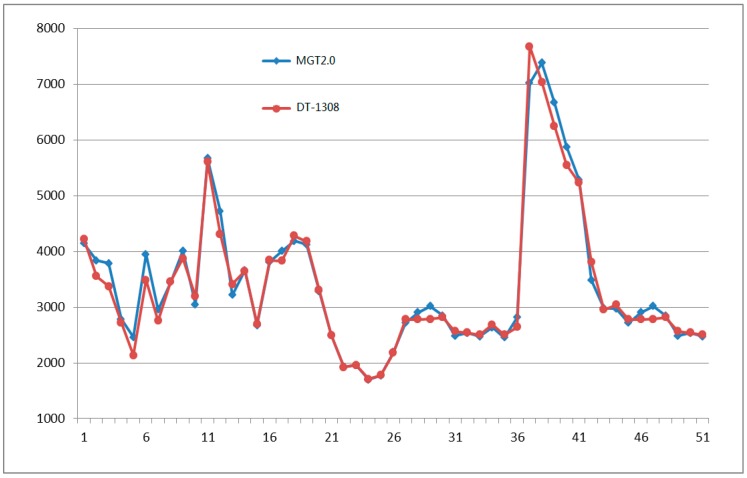
Fifty light intensity (Lx) experimental results by MGT2.0 (marked by **blue** line) and DT-1308 (marked by **red** line).

**Figure 14:**
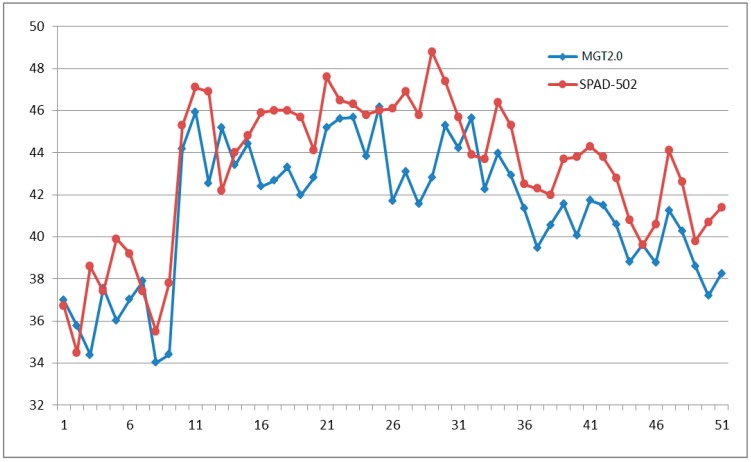
Fifty chlorophyll relative content (SPAD) experimental results by MGT2.0 (marked by **blue** line) and SPAD-502 (marked by **red** line).

In the experimental environment, the system ran continuously for more than 48 h, and the measured results were documented once every 10 min. The system provided 50 results randomly of environmental temperature, environmental humidity, light intensity, and chlorophyll relative content of crop leaves are shown in Figure 11, Figure 12, Figure 13 and Figure 14 respectively.

Figure 11 shows the random continuous 50 experimental temperature results and the temperature results of AR837 digital temperature and humidity instrument. The maximum absolute error is +2.1 °C, the average relative error is 2.84%, and the linear correlation coefficient *R* is 0.9606.

Figure 12 shows the random continuous 50 experimental humidity results and the humidity results of AR837 digital temperature and humidity instrument. The maximum absolute error is +9% RH, the average relative error is 11.21%, and *R* is 0.9980.

Figure 13 shows the random continuous 50 experimental light intensity results and the measurement results by DT-1308 illumination meter. The maximum absolute error is +10.30 Lx, the average relative error is 1.41%, and *R* is 0.9930.

Figure 14 shows the chlorophyll relative content on 50 samples of maize leaves at jointing stage of experimental system and SPAD-502. The maximum absolute error is −5.99 SPAD, the average relative error is −5.36%, and *R* is 0.9945.

The minimum linear correlation coefficient *R* is 0.9606 in air temperature, indicating a high degree of linear correlation between the experimental results by MGT2.0 and other single function measuring instruments used commonly. According to the maximum absolute error and the average relative error, air temperature, and light intensity measurement results are in line with the requirements; the average relative error of the air humidity is 11.21%, bigger than ±5% of the measurement error of AR837 digital temperature and humidity instrument, which is mainly due to changing of the air temperature, air pressure, and wind; the large measuring error of chlorophyll relative content of crop leaves mainly depends on the variation of structure and composition of plant leaves, the thickness of plant leaf has certain effects on the measurement, but the measurement accuracy of many simple repeated measures is less than ±1.0 SPAD. In the future, we will improve the portable multi-function farmland information collection system so that it has a wider range of applications and higher measurement accuracy.

## 4. Conclusions

Portable multi-function farmland information collection system realizes the function of portable and mobile measurement of multiple parameters information, which include the growth information of crops and the necessary environmental information. The characteristics of the system are summarized as follows:

(1) The farmland information collection device can measure multi-parameter information (chlorophyll relative content of crop leaves, location information, environmental temperature, environmental humidity, light intensity, and so on.) in a small agricultural environment, which provides the basic data for the automation management of agricultural production. STM32F103VET6 and μC/OS II V2.86 guarantee the system’s real-time ability. It could be carried by the farmer/technician on scheduled sampling trips for collecting the field information of corn or wheat crops in a small agricultural environment.
(2) UM220-III is used to realize the positioning based on BDS/GPS dual-mode system, which can obtain the real-time position information quickly and accurately, and it should be corresponding to the farmland information, so that agricultural technicians can understand the growth status of crops and environment information in different geographic locations in real time.
(3) CDMA wireless communication is used to realize the real-time remote transmission, which is convenient to remotely view and manage the collected data, and it solves the issues of time lag and inconvenience of data storage.

## Figures and Tables

**Table 1:** The basic requirements for the measurement parameters.

Parameters	Unit	Range	Accuracy (Average Relative Error)
Chlorophyll relative content	SPAD	−9.9–199.9	±5.5% (At the temperature of 25 °C)
Air temperature	°C	−30–50	±3.0%
Air humidity	% RH	0–99	±5.5% (At the temperature of 25 °C)
Light intensity	Lx	50–40,000	±3.5%
Soil temperature	°C	−30–50	±2.5%
Soil humidity	% RH	0–99	±5.5% (At the temperature of 25 °C)

**Table 2:** Parameters of SMET-2.

Parameters	Water Content	Temperature	Electronic Conductivity
Range	0%–100%	−40–85 °C	0–10,000 µs/cm (25 °C)
Unit	%	°C	µs/cm
Accuracy	±3% (0%–53%) ±5% (>53%)	<0.3 °C	2% FS
Interchangeable accuracy	<3%	<0.4 °C	<2% FS
Error of repetition	<2%	<0.2 °C	<2% FS
Electrical response time	1 s	1 s	1 s
Stabilization time	<2 s	<2 s	<2 s
Principle	FDR, Temperature and electrical conductivity correction	Heat sensitive element, nonlinear correction	Electrical conductivity measurement, temperature compensation

**Table 3:** Parameters of UM220-III.

Parameters	Values
Frequency	Beidou B1; GPS L1
First positioning time	Cold boot: 30 s; Hot boot: 1 s
Data update rate	1 Hz
Positioning accuracy (RMS)	Dual system: 2.5 m CEP; SBAS: 2.0 m CEP
Speed accuracy (RMS)	GPS/GNSS: 0.1 m/s; Beidou: 0.2 m/s
1 PPS	Support

**Table 4:** Parameters of SIM2000C.

Parameters	Values
Power supply	3.4–4.4 V
Power saving	In sleep mode, the current consumption is as low as 2 mA
Band	Single-band: 800 MHz (BC0)
Data transmission	CDMA 1×
	Downlink: Max 153.6 kbps
	Upload: Max 153.6 kbps
Temperature range	Normal operating temperature: −40–85 °C
	Storage temperature: –45–90 °C
Short message	Point to point MO and MT, text and PDU modes; support for ASCII and UNICODE; SMS storage: RUIM cards or modules
CDMA protocol	Protocol IS95-A/B:MSandBTS; IS-96A: coding of speech signals; IS-98A: base mobile station; IS-126: phonological loop; IS-637: short message service; IS-707: data services; IS-657: packet data
RUIM card interface	Supported RUIM card: 1.8 V, 3 V
Serial port	Supports standard full-featured serial port; fixed transmission rate is 115,200 bps, send commands and data through the serial port; support RTS/CTS hardware flow control, open or close through the software; programmable parameters
